# Palm is expressed in both developing and adult mouse lens and retina

**DOI:** 10.1186/1471-2415-5-14

**Published:** 2005-06-21

**Authors:** Meryl Castellini, Louise V Wolf, Bharesh K Chauhan, Deni S Galileo, Manfred W Kilimann, Ales Cvekl, Melinda K Duncan

**Affiliations:** 1 Department of Biological Sciences, University of Delaware, Newark, DE 19716 USA; 2 Depts. of Ophthalmology and Visual Sciences and Molecular Genetics, Albert Einstein College of Medicine, Bronx, NY 10461 USA; 3 Department of Cell and Molecular Biology, Uppsala University, S-75124 Uppsala Sweden; 4 Department of Pathology, Anatomy, and Cell Biology, Thomas Jefferson University, Philadelphia, PA 19107 USA; 5 Developmental Biology Division and Department of Ophthalmology, Children's Hospital Research Foundation, 3333 Burnet Avenue, Cincinnati, OH 45229 USA

## Abstract

**Background:**

Paralemmin (Palm) is a prenyl-palmitoyl anchored membrane protein that can drive membrane and process formation in neurons. Earlier studies have shown brain preferred *Palm *expression, although this protein is a major water insoluble protein in chicken lens fiber cells and the *Palm *gene may be regulated by Pax6.

**Methods:**

The expression profile of Palm protein in the embryonic, newborn and adult mouse eye as well as dissociated retinal neurons was determined by confocal immunofluorescence. The relative mRNA levels of *Palm, Palmdelphin (PalmD) *and *paralemmin2 (Palm2) *in the lens and retina were determined by real time rt-PCR.

**Results:**

In the lens, Palm is already expressed at 9.5 dpc in the lens placode, and this expression is maintained in the lens vesicle throughout the formation of the adult lens. Palm is largely absent from the optic vesicle but is detectable at 10.5 dpc in the optic cup. In the developing retina, Palm expression transiently upregulates during the formation of optic nerve as well as in the formation of both the inner and outer plexiform layers. In short term dissociated chick retinal cultures, Palm protein is easily detectable, but the levels appear to reduce sharply as the cultures age. *Palm *mRNA was found at much higher levels relative to *Palm2 *or *PalmD *in both the retina and lens.

**Conclusion:**

*Palm *is the major paralemmin family member expressed in the retina and lens and its expression in the retina transiently upregulates during active neurite outgrowth. The expression pattern of Palm in the eye is consistent with it being a Pax6 responsive gene. Since Palm is known to be able to drive membrane formation in brain neurons, it is possible that this molecule is crucial for the increase in membrane formation during lens fiber cell differentiation.

## Background

The retina and lens form from the neural tube and head ectoderm respectively. Despite these different origins, the development of the mature eye requires mutually inductive interactions between these two cell layers [1]. Further, in many cases, the lens and retina express the same developmentally important transcription factors [2-6]. In addition, a number of studies have identified the expression of proteins with known roles in neuronal function in the lens [7-12] and proteins important in lens function in the retina [13,14]. This may partially be due to the need of both retinal neurons and lens fiber cells to develop elaborated plasma membranes for their function [15-17].

Pax6 is a paired and homeodomain containing transcription factor that is required for the formation of the lens placode from the head ectoderm [18]. Specific loss of *Pax6 *expression from retinal progenitor cells results in the conversion of all retinal cell types to amacrine interneurons [19] and lens epithelial cells heterozygous for a *Pax6 *mutation preferentially differentiate into lens fiber cells [20]. Overexpression of the canonical form of Pax6 in lens fiber cells (Pax6 con transgenics) results in cataracts typified by incomplete lens fiber cell elongation and denucleation, instability of the transcription factor c-Maf and a drastic downregulation of β*B1-crystallin *expression [21] while overexpression of the Pax6 (5a) splice form also results in cataracts without the changes in cMaf stability [22]. Microarray analysis was previously performed on lenses from both Pax6 (con) transgenics and mice heterozygous for a *Pax6 *null allele and 13 genes were found to be upregulated in the transgenics and downregulated in the heterozygous knockout mice [23].

One of these genes, *paralemmin (Palm)*, encodes a protein present at the plasma membrane in axons, dendrites and perikarya of differentiating neuronal cell lines, and at high levels in the processes of the cerebellar molecular layer [24]. Further, this gene is downregulated in lenses overexpressing the Pax6(5a) splice variant [25] and the protein is detected in lens cells from both mice and chickens [25,26]. Overexpression of Palm in both neuronal and non-neuronal cell lines initiates the expansion of the plasma membrane and the development of extended processes and microspikes which is dependent on Palm targeting to the cytoplasmic face of the plasma membrane via a palmitoyl group covalently linked near the protein's C-terminus [24,27].

Here we investigate the distribution of Palm in the developing lens and retina, and compare its mRNA levels with two other members of the paralemmin family, *paralemmin-2 (Palm-2) *and *palmdelphin/paralemmin-like (PalmD) *[28,29].

## Methods

### Animals

All experiments using animals were approved by the both the University of Delaware and Albert Einstein College of Medicine Institutional Animal Care Committees and conform to the ARVO statement for the Use of Animals in Ophthalmic and Vision Research. C57Bl/6 mice were generated in-house from breeding stock obtained from Harlan Sprague Dawley (Indianapolis, IN). CD-1 mice were obtained directly from Charles River Laboratories (Wilmington, MA). Embryonic mice were staged by designating noon of the day on which a semen plug was observed in the dam as 0.5 days post-coitum (dpc). Postnatal mice were staged by designating the day of birth as 1 day postnatal (DPN). All mice were maintained in a 12-hour light/dark cycle at 21–24°C and were given food and water ad libitum.

### Immunofluorescent detection of Palm in tissue sections

Palm was detected by indirect immunofluorescence following the protocol previously described [30]. Briefly, tissue or embryos were excised from C57Bl/6 mice, embedded in tissue freezing media (TFM, Triangle Biomedical Sciences, Durham, NC) and sectioned at 16 μM on a Leica CM 3050 S Cryostat (Leica, Deerfield, IL). Sections were mounted on Colorfrost-plus™ slides (Fisher Scientific; Pittsburgh, PA), fixed in ice-cold acetone:methanol (1:1 vol/vol) for 15 minutes, dried and blocked with 1% BSA in phosphate buffered saline (PBS), pH 7.4. The blocking solution was removed and the sections incubated with a 1:150 dilution of rabbit polyclonal anti-Palm antibody [24] in 1% BSA-PBS for one hour at room temperature. The bound primary antibody was detected with AlexaFluor 568 goat anti-rabbit IgG (Molecular Probes, Inc. Eugene, OR) and cell nuclei were detected by counter-staining with TO-PRO-3 (1:3000 dilution in 1% BSA-PBS; Molecular Probes, Inc). Negative controls consisted of parallel staining experiments that omitted the primary antibody. Images were captured on a Zeiss LSM 510 Confocal Microscope configured with an Argon/Krypton laser (488 nm and 568 nm excitation lines) and Helium Neon laser (633 nm excitation line)(Carl Zeiss Inc, Göttingen, Germany).

### Transfections and reporter assays

Four copies of the Pax6-binding site previously identified in the human PALM promoter [25] were cloned into E4TATA-pGL3 [31] using a synthetic double stranded oligonucleotide 5'-ctagGGCT**ACTTTCACTCTGCGATGGCA**GAGCAGGGCT**ACTTTCACTCTGCGATGGCA**GAGCA-3'. Nucleotides containing Pax6-binding sites are in bold and nucleotides used for subcloning are indicated by lower case letters. Transient transfection assays were performed in 293T cells, which do not express endogenous Pax6 proteins, as described earlier [32].

### Immunofluorescent detection of Palm in cultured chick retina

Fertile White Leghorn eggs were obtained from the Department of Animal and Food Sciences at the University of Delaware and kept in a humidified, forced-draft incubator until embryonic day (E) 7. Retinas were dissected in calcium and magnesium-free saline solution (CMF). The neural retina was separated from the pigmented epithelium with fine forceps. The neural retina was minced with fine scissors and incubated in 0.25% trypsin in CMF for 20 minutes at 37°C. Retinas were dissociated into single cells by trituration with a Pasteur pipet in a 0.3 mg/ml soybean trypsin inhibitor/ 0.03 mg/ml DNaseI in Medium 199 (Cellgro, Herndon, Virginia). Cells were plated at a density of 5 × 10^5 ^cells / 12 mm diameter round glass coverslip in wells of a 24-well plate in one milliliter of Medium 199 (Cellgro) supplemented with 10% fetal bovine serum. Retina cultures were kept in a standard humidified culture incubator with 5% CO_2_.

Two days or one week after plating, cultures were fixed in 1% paraformaldehyde in PBS pH 7.4 for 30 minutes and then rinsed in PBS. Cells were then incubated for approximately 1 hour in a mixture of 1:200 rabbit polyclonal anti-chicken Palm [26] and 1:2 mouse monoclonal anti-neurofilament (RT-97) hybridoma supernatant (Developmental Studies Hybridoma Bank, Iowa City, IA; [33,34]) in PBS supplemented with 5% normal goat serum (NGS) and 0.03% Triton X-100 (TX-100). Cultures were rinsed in PBS and then incubated for approximately 1 hour in a mixture of 1:200 Alexa 488-goat anti-rabbit and 1:200 Alexa 594-goat anti-mouse secondary antibodies (both from Molecular Probes, Inc., Eugene OR) in the PBS/NGS/TX-100 mixture. Cultures were then rinsed in PBS and coverslips were mounted on glass slides in a buffered glycerol mounting medium containing ρ-phenylenediamine to retard photo-bleaching. Cultures were observed and photographed using a Nikon Microphot FX epifluorescence microscope equipped with a Nikon DXM-1200 CCD camera. Red and green channel images were merged using Adobe Photoshop.

#### Real Time RT-PCR

Tissue microdissected from the lens, cerebellum and telencephalon was stored in RNA later (Qiagen, Valencia, California). Total RNA from the lens, cerebellum and forebrain of newborn CD-1 mice was isolated using the RNeasy Protect Mini Kit (Qiagen). Retinal P0, P4 and P22 RNA was kindly provided by Drs. Mike Dorrel and Kenneth Mitton, respectively. DNaseI digestion was performed during RNA isolation with RNase-Free DNase Set (Qiagen). The RNA was quantified with an Agilent 2100 Bioanalyzer and first strand cDNA was then synthesized using 5 μg of RNA, Oligo(dT)_12–18 _primer and Superscript II RT (Invitrogen, Carlsbad, California) as per manufacturer's instructions. The cDNA was diluted 1:10 and PCR reactions were conducted using 2 μl of cDNA, 50 nm of forward and reverse primers, and 2X SYBR Green PCR Master Mix (Applied Biosystems, Foster City, California). Amplification of the cDNA was performed using a 7900 HP Applied Biosystems Real Time PCR machine. The cDNA was initially denatured at 94°C for 5 minutes, followed by 45 cycles of 94°C for 10 seconds, annealing at 60°C for 20 seconds, and extension at 72°C for 30 seconds. A final extension at 72°C for 5 minutes was then conducted. Each gene was amplified nine times (three times as triplicate experiments). The primers used with Ensembl or NCBI accession numbers follow: *Palm (*ENSMUSG00000035863) (5' -AGCAGGCAGAGATTGAGAGC-3' and 5' -AGCCAGCGTTCCCTCAGT-3'); *Palm2 *(NM 172868) (5' -CGCAGGCAGTCTGAAGAAG-3' and 5' -TTTCGAGCGCTTGTATTTCC-3'); *PalmD *(ENSMUSG00000033377) (5' -AGTAGCTGGAGACGGGACTG-3' and 5' -CACGGCTCTCAGATCACCTT-3'). The housekeeping genes *β2-microglobulin*, *B2M *(ENSMUSG00000033376) (5' -TGGTGCTTGTCTCACTGACC-3' and 5' -TATGTTCGGCTTCCCATTCT-3'); *Hypoxanthine-guanine phosphoribosyltransferase*, *HPRT *(ENSMUSG00000025630) (5' -GTTGTTGGATATGCCCTTGA-3' and 5' -GGCTTTGTATTTGGCTTTTCC-3'): and *succinate dehydrogenase*, *SDHA *(ENSMUSG00000021577) (5'-GAGGAAGCACACCCTCTCATA-3' and 5' -GCACAGTCAGCCTCATTCAA-3') were used for normalization of gene expression levels. Each primer set was designed using Primer3 [35] and specificity verified by NCBI Blast [36]. Standard PCR was then performed to verify amplification of a single PCR product bearing the correct size. The dissociation curve of each PCR amplicon was analyzed using ABI PRISM SDS 2.0 and revealed a single peak, indicating specific PCR amplification [37].

The mRNA levels were normalized to the internal housekeeping gene, *B2M *and the change in C_t _values for each gene (ΔC_t_) were determined according to the standard method [38,39]. The standard deviation calculated for each sample was less than 5% and was therefore not shown in Figure 5. The primers used had similar efficiencies for amplification as determined by serial dilution experiments [38].

## Results and discussion

Previously, we determined that Palm gene expression is downregulated in lenses from mice lacking one copy of the *Pax6 *gene [25] and upregulated in lenses overexpressing Pax6 [23]. Since potential Pax6 binding sites were identified upstream of the transcriptional start site of *Palm *[25], *Palm *may be a direct Pax6 target gene. Thus, we undertook a developmental expression study of Palm in the eye to assess the extent that its expression overlaps that of Pax6.

**Figure 1:**
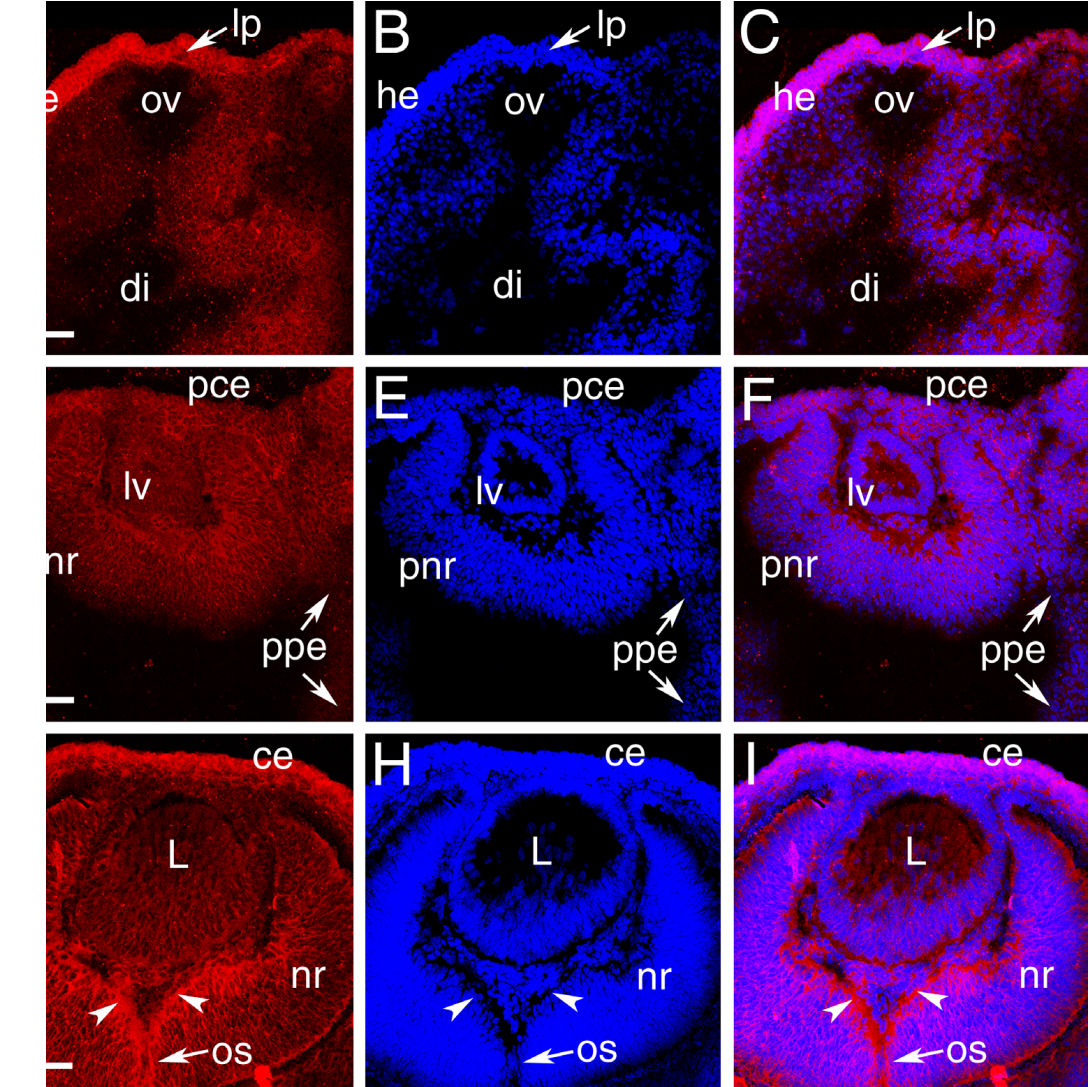
Localization of Palm protein during early mouse eye development. A-C, 9.5 dpc; D-F, 10.5 dpc; G-I, 12.5 dpc; A,D,G Palm; B,E,H cell nuclei stained with ToPro3, C,F,I, merge; Abbreviations- lp, lens placode; ov, optic vesicle; he, head ectoderm; di- lumen of the diencephalon; pce- presumptive corneal epithelium; lv- lens vesicle; pnr- presumptive neural retina; ppe- presumptive retinal pigmented epithelium; L- lens; nr- neural retina; ce- corneal epithelium; os- optic stalk. Arrowheads denote staining in developing neuronal processes that will grow through optic stalk to form the optic nerve. All scale bars are 77 μm. red- Palm; blue-ToPro3 DNA stain.

At 9.5 dpc, Palm immunoreactivity is prominent in the head ectoderm overlying the optic vesicle that is fated to give rise to the lens and corneal epithelium with much lower, but detectable, levels of expression in the optic vesicle (Figure 1A,B,C). At 10.5 dpc, Palm protein is detected at relatively similar levels in the presumptive neural retina, retinal pigmented epithelium (RPE), corneal epithelium and lens vesicle (Figure 1D,E,F). This overlaps well with Pax6 expression in both the optic vesicle and developing lens placode/ vesicle in mice [18,40]. By 11.5 dpc, relative Palm levels have decreased in the presumptive RPE although staining is still detected in both the periocular mesenchyme and presumptive neural retina (data not shown). At 12.5 dpc, intense Palm immunoreactivity is detected at the vitreal surface of the neural retina (Figure 1G, H, I), corresponding to the formation of the ganglion cell processes that will migrate down the optic stalk to form the neural component of the optic nerve [41,42]. This only partially corresponds with Pax6 expression at this stage, since Pax6 expression has been reported in the developing RPE of 13 dpc embryos [43], although the RPE can produce pigment without Pax6 [4]. The presence of Palm in ganglion cell processes at this stage is interesting since Pax6 expression is noted in mature ganglion cells of the adult retina although Pax6 is detected in only a subset of 13 dpc neural retinal precursors [43].

**Figure 2:**
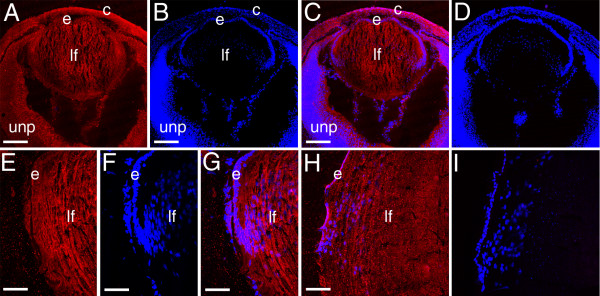
Localization of paralemmin protein in the mouse lens A-C 14.5 dpc; D 14.5 dpc negative control E-G One week post natal; H- 25 weeks postnatal. I- 25 weeks postnatal negative control A,E- paralemmin; B,F- cell nuclei stained with ToPro3; C, D, G, H, I- merge; Scale bars- A-C, 154 μm; D-G, 77 μm; red- paralemmin; blue-ToPro3 DNA stain.

In the developing mouse lens, Palm expression is seen at both epithelial and fiber cell membranes from 11.5 dpc and is maintained in these cells throughout adulthood (Figure 1D–I; Figure 2). The presence of Palm in all lens cells early in development correlates well with the reported expression pattern of Pax6 in the embryonic lens [18]. During lens maturation, Pax6 expression decreases in lens fiber cells relative to the lens epithelium [21,22,44]. However, newborn rat lens fiber cells still maintain 12% of the levels seen in lens epithelium [45] although Pax6 mRNA levels are 95 fold lower in aged human lens fibers [46]. Since Palm mRNA levels are decreased in lenses from Pax6 heterozygous mice [25] and upregulated in lenses overexpressing Pax6 in lens fiber cells [23], it is plausible that Palm expression is either directly responsive to Pax6 or controlled by genes in the same pathway.

**Figure 3:**
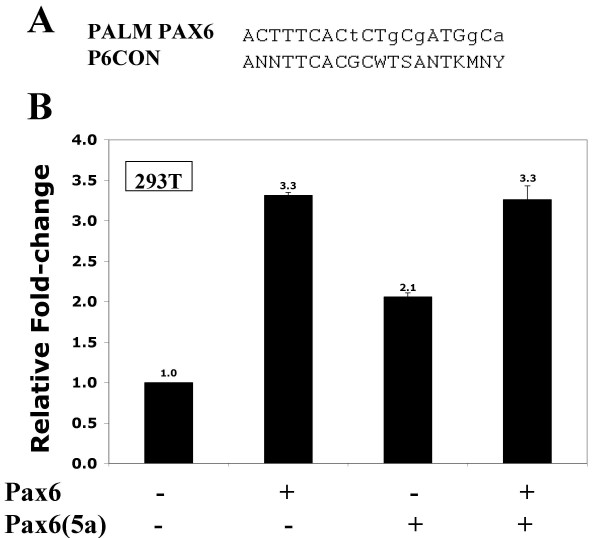
Pax6 proteins activate expression from a reporter consisting of four copies of a PAX6-binding site found in the putative 5' flanking sequence of the human PALM gene cloned upstream of the E4 basal promoter. (A) An alignment between the PAX6 site found in the PALM gene and a consensus paired domain Pax6-binding site, P6CON. Non-conserved nucleotides are shown in lower case letters. (B) Results of co-transfections in 293T cells. 200 ng of Pax6 and 25 ng of Pax6(5a) expression plasmids were used as indicated per experiment. The data were normalized using *Renilla luciferase *[31] and are expressed as a relative ratio of promoter activity in the presence of Pax6 compared to the presence of empty vector, pKW10.

In order to test this proposition functionally, we cloned the Pax6 binding site previously identified in the 5'-flanking region of human *PALM *[25] in front of a basal promoter and performed transient transfections in 293T cells which lack endogenous Pax6 proteins [32]. Co-transfection of this reporter construct with Pax6 and Pax6(5a) expression vectors activated this artificial promoter 3.4- and 2.1-fold, respectively while addition of both expression vectors simultaneously yielded a reporter activation similar to that of the Pax6 expression vector alone (Figure 3). These levels of Pax6 mediated activation are comparable to those typically obtained in transient transfections with Pax6 responsive promoters [44,47,48]. From these data, it appears likely that the human *PALM *promoter contains a Pax6-binding site functionally able to interact with both Pax6 and Pax6(5a) consistent with the upregulation of *PALM *expression in transgenic mice overexpressing Pax6 in the lens and reduced expression of Palm in Pax6 heterozygous lenses [23,25,49]. However, the functional significance of this Pax6 site in the context of the *PALM *gene is more difficult to ascertain since neither the transcriptional start site nor the functional minimal promoter of *PALM *have been experimentally investigated. Further studies of *PALM/Palm *promoters are necessary to fully establish their direct regulation by Pax6 proteins.

**Figure 4:**
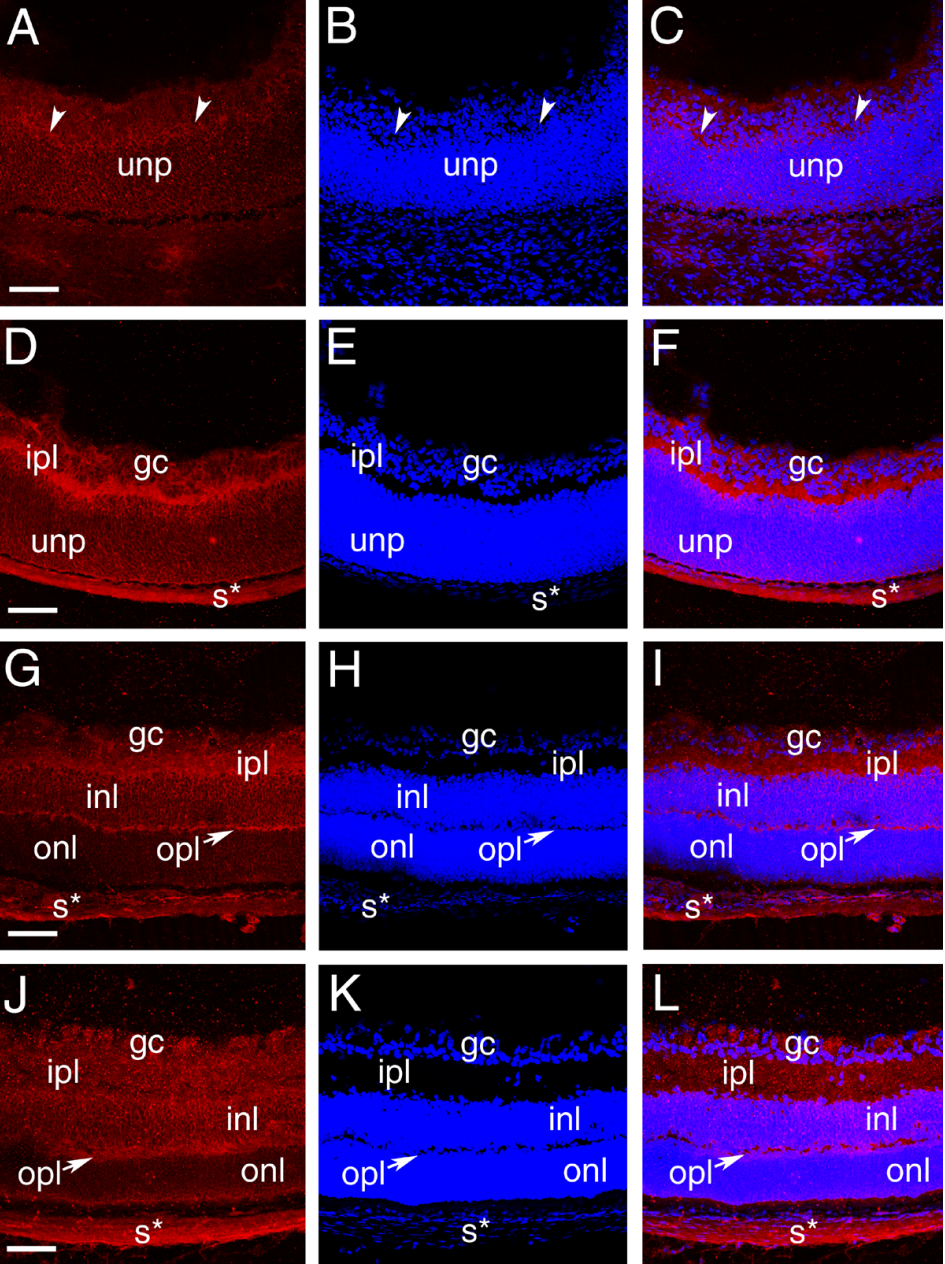
Localization of paralemmin protein during mouse retinal development A-C, 16.5 dpc, arrowheads- emerging inner plexiform layer; D-F 1 day pn; G-I 1 week pn; J-L 2 week pn; A,D,G,J- paralemmin; B,E,H,K- cell nuclei stained with ToPro3; C,F,I,L- merge; Abbreviations- unp- undifferentiated retinal precursors; gc- ganglion cell; ipl- inner plexiform layer; s*- background staining in the sclera; inl- inner nuclear layer; opl- outer plexiform layer; onl- outer nuclear layer. All scale bars are 77 μm. red- paralemmin; blue-ToPro3 DNA stain.

In the developing retina, the intense Palm staining seen in elongating ganglion cell axons at 12.5 dpc downregulated markedly by 14.5 dpc as the development of these processes completes [41] (data not shown). At 16.5 dpc, Palm immunoreactivity is maintained at moderate levels in the cell bodies of both differentiating ganglion cells and undifferentiated neural precursors, but appears slightly stronger in the first morphologically distinguishable axons of the developing inner plexiform layer (ipl) which is composed of cell processes of the neurons of the inner nuclear layer and ganglion cells [50](Figure 4A,B,C). At birth, Palm levels are upregulated in the developing inner plexiform layer (inl) which is in the process of rapid expansion (Figure 4D,E,F). As the development of ipl proceeds, the intensity of Palm staining in this layer drops to that seen in the cell bodies of the ganglion cell and inl (Figure 4G–L). While not as dramatic, localized expression is seen in the developing outer plexiform layer (opl) processes at 1 week pn (Figure 4G–I), although both at that time and in the adult (Figure 4J–L), much less Palm staining is seen on the photoreceptor cell bodies of the outer nuclear layer then in any other retinal layer. Notable, Pax6 expression persists in both retinal ganglion cells and the inner nuclear layer into adulthood, correlating well with the expression pattern of Palm in this tissue [43].

**Figure 5:**
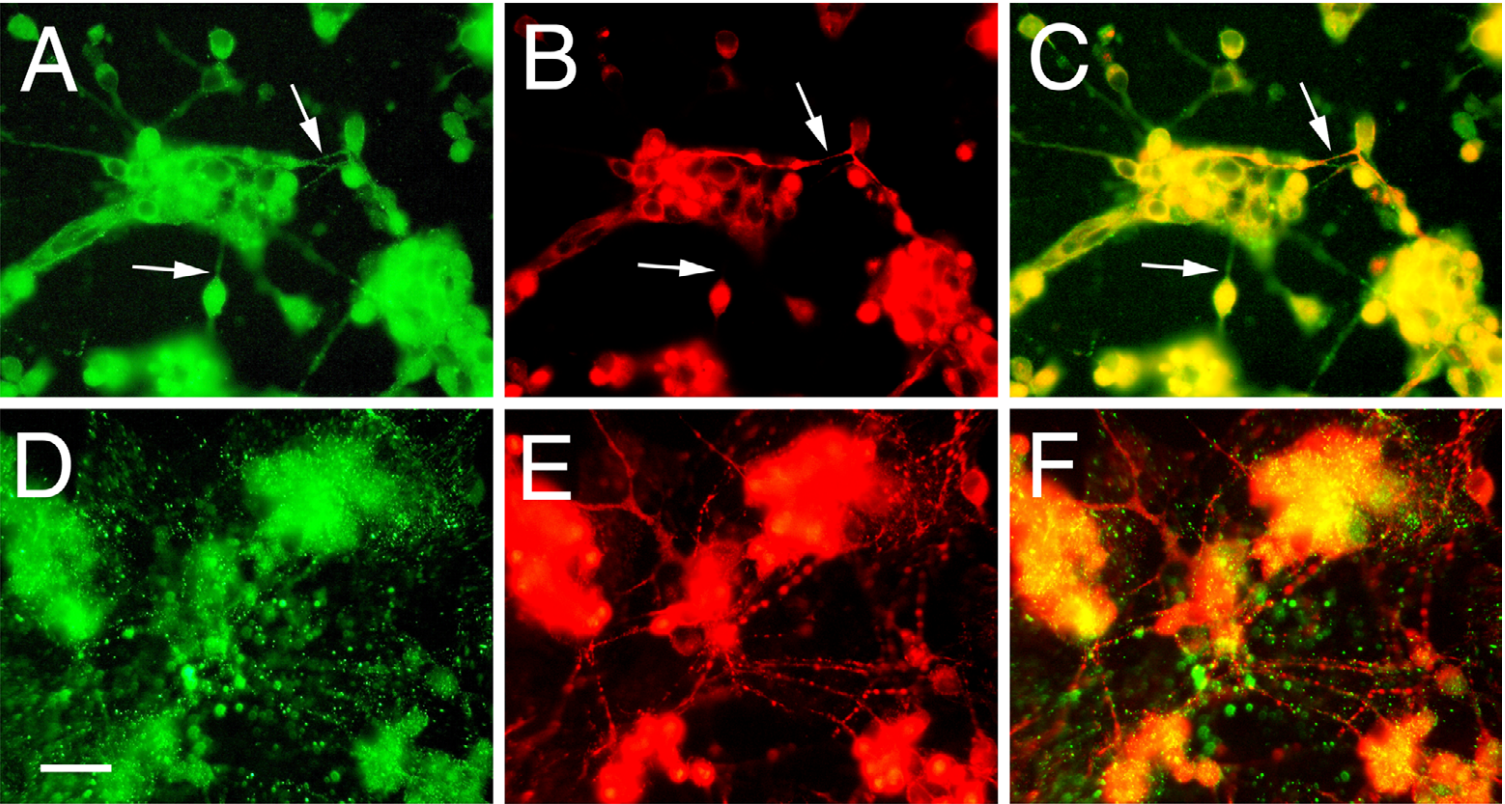
Expression and localization of Palm in chick retinal cultures. Cultures were immunostained with polyclonal anti-Palm (A, D) and RT-97 anti-neurofilament (B, E) antibodies after 2 (A-C) or 7 (D-F) days in culture. For each pair, the merged images are shown in C and F. After 2 days in culture, Palm is present on most cells at cell borders as well as intracellular puncta (A). Fine processes resembling axons (arrows) that are sometimes positive for RT-97 (B) are also labeled. After 7 days in culture, Palm staining appears punctate but more diffuse (D), and does not appear to be localized on the numerous long processes stained with RT-97 (F). Bar in D, 25μm. Green- Palm; Red-RT-97;

In neuronal cell lines, Palm was previously detected at the cell membrane of the cell body and developing axons as well as in a granular localization intracellularly. *In vivo*, Palm co-purifies with chick brain synaptic plasma membranes consistent with its palmitoylation [24]. While the staining pattern of Palm in the developing mouse retina is consistent with this membrane localization, we wanted to confirm this in dissociated retinal cultures. The neural retina of the E7 chick is at a period of extensive neurogenesis, migration, and process formation *in vivo*, especially of ganglion cells [51-53]. This ability to extend neurites is also manifest in cultures made from this age retinal tissue [54,55]. Chick retinas were dissociated, plated and stained for Palm either 2 days or 7 days after plating. After 2 days in culture (Figure 5A–C), Palm appears expressed by most cells and is evident at the plasma membrane and as intracellular puncta. Fine processes resembling axons (arrows) that sometimes stain with the anti-neurofilament antibody RT-97 [33] are also positive for Palm immunoreactivity. After 7 days in culture (Figure 5D–F), Palm staining appears punctate but more diffuse in the cell body, and does not appear to be localized on the numerous long processes stained for neurofilament. Thus, like in the mouse retina *in vivo*, Palm is detected in retinal cultures undergoing active process formation while it is less evident in mature cells, which are undergoing less process extension.

*Palm *is a member of a multigene family consisting of two other family members, *paralemmin-2 (Palm-2) *and *palmdelphin/paralemmin-like (PalmD/PalmL) *[28,29]. Palm2 shares 37% amino acid identity with Palm and like Palm has a C-terminal CaaX motif that could potentially be prenylated. However, the Palm2 gene is alternatively spliced and not all variants contain the prenylation motif. PalmD is 23% identical to Palm but generally lacks a C-terminal prenylation motif although rare splice variants have an alternative C-terminus containing a prenylation motif similar to Palm. Experimentally, the majority of PalmD is cytoplasmic and does not co-purify with plasma membrane fractions [28,29]. Since Palm is potentially able to modulate plasma membrane growth in the lens, retina and brain, while Palm2 and PalmD are of related sequence, we performed quantitative rt-PCR to compare the relative expression levels of all three paralemmin family members in the lens, retina, cerebellum and forebrain.

The ratio between the housekeeping genes tested, *B2M*, *HPRT *and *SDHA*, in the different tissues analyzed was found to range between 0.98–1.04. Since the ratio of an ideal internal control between various tissues would be 1 and the variability of each of our internal normalizing genes between the various tissues assayed was low, we normalized our data to one housekeeping gene, *B2M *[39,56].

**Figure 6:**
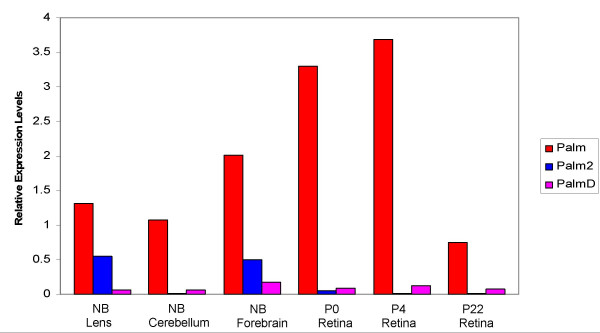
Relative levels of *Palm*, *Palm2 *and *PalmD *transcripts in the lens, cerebellum forebrain and retina. All data are expressed as a relative to the amount of *B2M *in the sample.

In the lens, cerebellum, forebrain and retina, *Palm *transcripts are significantly more abundant relative to *B2M *than those of either *Palm2 *or *PalmD *(Figure 6). Notably, *Palm *mRNA is more abundant in retinas isolated shortly after birth compared to the adult retina, correlating well with the expression of Palm protein detected by immunohistochemistry. *Palm *is alternatively spliced, and previous western blot analysis of mouse lens protein detected the 60 kDa form of paralemmin [25] which translates from mRNA lacking exon 8 [24]. Parallel qt-PCR analyses of the lens and retina for *Palm *transcripts harboring exon 8 only detected low levels of this splice variant in all cases (data not shown) which would be translated into a 80 kDa protein. In the lens and forebrain, appreciable *Palm2 *expression was detected (C_t _values of about 21.5) while *Palm2 *levels are relatively low in all post natal retinal samples tested (C_t _values of about 28.5). *PalmD *transcripts were usually present at low levels in the tissues examined with C_t _values of about 26. Co-expression of *Palm *with *Palm2 *in tissues examined will aid to the interpretation of gene targeting studies of this family of genes.

## Conclusion

The lens and retina express paralemmin during development with its transient upregulation during the formation of optic nerve and formation of both plexiform layers. Further, the putative *PALM *promoter contains a functional Pax6 binding site and the developmental expression pattern of Palm in the eye generally correlates well with that reported for Pax6, leading credence to the idea that *Palm *is a Pax6 directly-regulated gene.

## Abbreviations

dpc, days post coitum; PBS- phosphate buffered saline; inl, inner nuclear layer; onl; outer nuclear layer; opl, outer plexiform layer; ipl; inner plexiform layer; pn, post-natal; rpe, retinal pigmented epithelium.

## Competing interests

The author(s) declare that they have no competing interests.

## Authors' contributions

MC carried out all of the immunohistochemical studies on tissue and was involved in the initial drafting of the manuscript. LVW carried out all of the quantitative rt-PCR assays and BKC performed the transfection assays. DSG analyzed PALM expression in chick retinal cultures and MWK was involved in the experimental design and its interpretation. AC conceived of the molecular experiments and participated in their design and interpretation. MKD imaged all of the immunohistochemical data, was involved in its interpretation and drafted the manuscript at all stages of the submission process.

## Pre-publication history

The pre-publication history for this paper can be accessed here:


